# Family physicians’ experiences with an electronic medical record-integrated family history collection strategy: a qualitative study

**DOI:** 10.3399/BJGPO.2025.0051

**Published:** 2026-05-20

**Authors:** Sakina Walji, Tutsirai Makuwaza, Erin Bearss, Sahana Kukan, Babak Aliarzadeh, Judith Allanson, Michelle Greiver, Eva Grunfeld, Karuna Gupta, Ruth Heisey, Noah Ivers, Doug Kavanagh, Raymond Kim, Michelle Levy, Rahim Moineddin, Shawna Morrison, Maria Muraca, Donatus Mutasingwa, Mary Ann O'Brien, Joanne Permaul, Frank Sullivan, Brenda J Wilson, June C Carroll

**Affiliations:** 1 Department of Family and Community Medicine, University of Toronto, Toronto, Canada; 2 Sinai Health System, Toronto, Canada; 3 Children’s Hospital of Eastern Ontario, Ottawa, Canada; 4 North York General Hospital, Toronto, Canada; 5 Markham Stouffville Hospital, Markham, Canada; 6 Women's College Hospital, Toronto, Canada; 7 University Health Network, Toronto, Canada; 8 School of Medicine, University of St Andrews, St Andrews, UK; 9 Division of Population Health and Applied Health Sciences, Memorial University of Newfoundland, St John's, Canada

**Keywords:** family medicine, family history, personalised medicine, electronic health records, primary health care, qualitative research

## Abstract

**Background:**

A complete, up-to-date family history (FH) is imperative in primary care to identify those at increased risk of heritable conditions who may benefit from personalised screening and management. Complete FH is rarely documented in the electronic medical record (EMR).

**Aim:**

To understand family physicians’ (FPs’) experiences of an EMR-integrated FH strategy.

**Design & setting:**

A descriptive qualitative study was conducted using one-to-one interviews to assess a FH strategy. Primary care teams, affiliated with University of Toronto Practice-Based Research Network in Ontario, Canada, were randomly selected. The participants were FPs from three sites that implemented the strategy.

**Method:**

Telephone interviews were undertaken with FPs. Thematic analysis was used for identifying, analysing, and reporting patterns in the data. An iterative process was used, with modification of interview and coding guides as new themes emerged.

**Results:**

A total of 14 out of 15 FPs were interviewed. The following six major themes were identified: 1) FH informs hereditary risk and enables tailored patient care; 2) routine, intentional FH collection by patients and FPs is essential; 3) FH collection supports meaningful patient–FP discussions and quality care; 4) point-of-care tools enhance FP awareness and knowledge; 5) success is supported by patient engagement and EMR integration; and 6) tailored approaches are needed to improve acceptability.

**Conclusion:**

Physicians expressed the importance of routine FH collection and its implications for clinical management. Factors contributing to the strategy’s success included being patient-initiated and medical record integration.

## How this fits in

A complete and up-to-date family history (FH) is essential in primary care (PC) to identify individuals at increased risk for heritable conditions, enabling personalised screening and treatment. However, FH collection is inconsistent, with low documentation rates in electronic medical records (EMRs), leaving many high-risk individuals unidentified. Family physicians (FPs) recognise FH as vital but cite barriers such as time constraints, competing priorities, and challenges in obtaining reliable information. This study evaluated FPs' experiences with an EMR-integrated FH collection strategy using electronic patient questionnaires and clinical support tools to assess engagement and inform future implementation in PC.

## Introduction

A complete and up-to-date FH is imperative in PC to identify individuals at increased risk for heritable conditions and to enable personalised screening and treatment.^1,2^ The US Preventive Services Task Force recommends thorough FH collection to identify hereditary syndromes^3^ and the Centers for Disease Control and Prevention highlight several conditions where FH can guide risk stratification and care.^4^ For example, individuals with a genetic predisposition to breast cancer may reduce their risk by 90%–95% through bilateral mastectomy.^5^

Despite its importance, FH is often incomplete or missing in the electronic medical record (EMR).^6,7^ One US study reported only 30% of PC patients had documented FH within the EMR,^8^ and direct observation of new patient encounters in PC revealed FH was discussed in only 51% of encounters.^8^ Thus, many individuals at increased risk may remain unidentified.^9–12^

Family physicians (FPs) value FH for its clinical and relational benefits, but find documenting and using FH in practice challenging owing to time constraints, limited genetic knowledge, lack of specialist access, and concerns about the reliability and utility of patient-provided information.^7,13–16^

Physicians report that improving the integration of FH into workflow and providing decision support may improve patient care.^17^

Despite its importance, FH collection in PC remains inconsistent and poorly integrated into clinical workflows.^18^ Evidence is limited on how FPs engage with FH collection strategies, particularly those embedded into EMRs.^19^ This study addressed that gap by exploring FPs’ experiences with an EMR-integrated FH collection strategy to identify factors that influenced its implementation. This study is part of a larger implementation-effectiveness trial evaluating the strategy’s impact on FH documentation.^20^ While the quantitative component evaluated documentation outcomes, this study specifically examined FP engagement.

## Method

### Study design

We undertook a descriptive qualitative study to assess FP experiences with a FH collection strategy. The Consolidated Framework for Implementation Research (CFIR) can be used to guide and assess intervention strategies;^21^ this framework was used to design our strategy. The strategy had the following four components: 1) education of patients about FH through email information and waiting room videos, and FPs through webinars. These were developed by the team, including a genetic counsellor with expertise in patient and healthcare professional education; 2) review of clinic schedules of participating FPs 2 weeks before upcoming appointments, with random selection of patients for invitation to complete the FH screening questionnaire (FHSQ), a validated seven-question patient-administered questionnaire (two additional questions added by the research team)^22^ (see Supplementary Information S1); 3) EMR integration of the FHSQ, with a message to the FP alerting them that the FHSQ had been completed and was available for review. Patients completed the FHSQ via a secure emailing platform integrated with the EMR system. On completion, the research associate (SK) received an automated notification. SK then sent a message to the FP indicating that the completed questionnaire was available for review. The FHSQ was not automatically entered into the patient’s electronic health summary; rather, it was delivered to FPs as a document within the EMR, similar to a laboratory result. FPs could then choose to incorporate relevant information into the health summary at their discretion; and 4) electronic clinical decision support through linked point-of-care tools. These were developed in collaboration with Genetics Education Canada: Knowledge Organization (https://geneticseducation.ca); a website that is dedicated to producing PC-focused genomics resources. The point-of-care tools provided risk assessment information and genetics referral criteria.

FPs had autonomy to manage collected FH as preferred and could discuss it with the patient at their upcoming visit if they wished. FPs were advised of available resources, such as the point-of-care tools that might help them in the management of FH information, and were free to use their professional judgement in when and how to manage the information presented to them.

### Setting and participants

The strategy was implemented over 6 months from September 2021 to June 2022 (exact start date varied across sites) with PC multidisciplinary team practices affiliated with the University of Toronto Practice Based Research Network (UTOPIAN),^23^ now re-formed as UPLEARN,^23^ and that used the Ocean emailing platform (https://www.oceanmd.com) and PS Suite EMR^24^ in Ontario, Canada. All participating sites were PC teams located in downtown or suburban Toronto. While patient populations and practice characteristics varied across sites, all used the same EMR system with the capability to send patient emails via a secure portal. These teams operate within Ontario’s publicly funded healthcare system and reflect the digital infrastructure and diversity typical of urban and suburban PC settings.

FPs were eligible for the qualitative study if they belonged to a practice that was implementing this strategy as part of the intervention arm of the implementation and effectiveness trial.^20^ All FPs participating in the qualitative study had experience with the FHSQ. Eligible participants were invited by email from the principal investigator (JCC).

### Data collection

Interviews took place between June 2022 and July 2022. One-on-one telephone interviews were conducted by an experienced qualitative research assistant (TM) using a semi-structured interview guide (see Supplementary Information S2) developed from research team experiences, study objectives, and literature.^25,26^ The interview guide was pilot tested with three FP team members. FPs were asked about their experiences and perceptions of the strategy including its value, clinical utility, and implementation. The guide was iteratively refined to probe emerging ideas. Interviews were audio-recorded, transcribed verbatim, and anonymised. Demographic information including age, sex, and years in practice was collected.

### Data analysis

Initially, three team members (JCC, SW, and TM) independently performed line-by-line open coding of interview transcripts and then met to discuss codes.^27^ After coding two transcripts, the group met to discuss a coding framework. Two more transcripts were reviewed by these three team members and after reaching agreement, we developed a coding framework used by TM to analyse the remaining transcripts.

Our positioning (JCC and SW) about the importance of FH was informed by professional values as practising FPs. TM conducted all interviews; she is a trained qualitative researcher and had no prior relationship with the participants. SW and JCC, both FPs with research backgrounds, were involved in line-by-line coding and thematic analysis. Their professional perspectives influenced interpretation, a fundamental and necessary component of qualitative analysis, with TM ensuring that the emerging themes remained grounded in the data and true to participants' accounts. Theme development was iterative and involved regular team discussions, including input from non-FP researchers, to challenge assumptions and enhance reflexivity.

Team members met to review the coded data and supporting verbatim quotes, then identified major emerging themes from the data. Information power of the major themes was deemed to be reached when further data from the interviews offered no new insights that contributed or challenged the understanding from the data.^28^

Coding disagreements were resolved through discussions. An inductive approach guided analysis, using the constant comparative method^29–31^ to sort and organise data. Memos and meeting notes created an audit trail to ensure transparency and rigour.^32^ Themes were developed by a smaller group (JCC, SW, and TM) then discussed with a larger group of authors including FPs and qualitative researchers (JCC, SW, TM, SK, BJW, EB, and MAO), then finally discussed with the full research team.

NVivo (version 12) software was used to store transcripts and manage the study data. We used the Consolidated Criteria for Reporting Qualitative Studies criteria for reporting qualitative research.

The CFIR helps assess strategy components that support successful implementation.^21^ It comprises the following five main domains: intervention characteristics; outer setting; inner setting; individuals involved; and implementation process. The CFIR was used to inform strategy design but not the interview guide or data analysis. It was applied post-analysis to assess which characteristics of the strategy contributed to its effectiveness.

## Results

Of 15 eligible participants, 14 completed interviews (Table 1). One participant did not respond to the invitation in time to be included.

**Table 1.** Participant characteristics

Characteristic	*n* (%)
**Age, years, mean (range)**	47.9 (35 to 69)
**Sex**	
Female	10 (71.4)
Male	4 (28.6)
**Years in practice**	
0–10	6 (42.9)
>10	8 (57.1)
**Practice type** ^**a**^	
Community	1 (7.1)
Academic	8 (57.1)
Combined	5 (35.7)

^a^Practice type was self-defined.

Transcript analysis identified six key themes. FH was valued as a crucial tool for assessing hereditary risk and supporting personalised care. Its routine collection and review by both patients and FPs were central. FH strategy fostered meaningful patient–physician conversations and was linked to perceptions of high-quality care. Point-of-care tools helped improve FPs’ knowledge and risk management. Success factors included active patient engagement and EMR integration. Tailored implementation approaches were seen as essential for broader acceptance. See Supplementary Box S1 for themes and sub-themes.

### FH informs hereditary risk and enables tailored patient care

Participants acknowledged the importance of FH for risk stratification and provision of personalised preventive care. In some instances, the newly identified FH prompted an EMR update or changes in patient management, such as earlier screening or genetics referral:

*‘I actually found it* [the FH strategy] *very useful. And it helped me identify a few important family history elements for certain patients that actually ended up changing management or triggering some investigations in consultation with genetics … And these are people that I had asked about family history before and these things didn’t come up*.*’* (Participant [P]003)

Participants commented on the importance of collecting FH beyond the FHSQ even if not associated with current evidence-based interventions. This might enable future interventions and give a broader picture of the family’s health:

*‘The fact that there’s no test doesn’t necessarily negate the potential value of the information to the patient, and/or in the future, should a test become available ... most people seem to want to, whether there is something to do or not, and sometimes it is to be able to inform the rest of their family.’* (P009)

### Routine, intentional FH collection by patients and FPs is essential

Participants noted that the strategy enabled systematic FH review. FPs lacked a consistent method for updating FH, relying on patient disclosure or presenting complaints, potentially resulting in new FH being overlooked. While most felt they knew their patients well, some discovered previously undisclosed or outdated FH:

*‘There were certainly a few patients where they disclosed something that I wasn’t aware of, either because it was new or it had just happened, even though it’s been a year or two prior. Or because, for whatever reason, when we did the family history the first time, it didn’t come up or I didn’t ask the question properly or they didn’t understand the question or what have you ... I’m sure that it resulted in an earlier mammogram or a change in cardiovascular risk assessment. Like I’m sure that it did provide information that changed this patient’s plan.’* (P001)

Systematic FH collection allowed FPs to revisit previously collected FH and determine if recommendations had changed:

*‘I have some patients who do have concerning family histories, have had genetic testing or haven’t qualified for genetic testing. But then to revisit that decision 10 years later let’s say with this tool* [strategy] *when there have been new developments and then it’s like well they didn’t qualify 10 years ago but actually now they do or something like that …. so that’s super useful.’* (P002)

Despite the unified opinion on the importance of FH data in patient management, there were differing experiences of usefulness of the strategy. While some FPs stated that FH was not routinely updated owing to competing priorities and changes to guidelines on frequency of preventive care visits, others said they were already aware of any new FH. One FP stated, *‘I didn't find it very useful, because I already had it in the chart’* (P006).

### FH collection supports meaningful patient–FP discussions and quality care

Encouraging patients to reflect on their FH and discuss it during clinical encounters improved patient education and engagement. FH discussion supported patient-centred care by facilitating active listening and meaningful conversations:

*‘It started a really important conversation with patients since they often don’t really understand what the implications of genetics are.’* (P002)

Routinisation of FH collection and meaningful subsequent discussions may have positive implications for preventive care and patient outcomes that extend beyond genetics-related issues by engaging patients in healthy behaviours and screening:

*‘I think it is potentially valuable in that it could provide an added impetus for patients to sort of engage in healthy behaviours, if they're aware of risks … ’* (P014)

Participants noted the strategy improved understanding of their patients’ context, such as emotional or psychological impacts from a loved one’s health, insights that might improve patient-centred care.

### Point-of-care tools enhance FP awareness and knowledge

FPs described that they were already aware of common inherited cancers and the appropriate risk-based screening tests. However, the strategy reinforced their existing knowledge and brought FH to the forefront of their mind:

*‘It* [strategy] *had me think a little bit more broadly about the association between different types of cancers. I always think about breast cancer and colon cancer, but there are some others that can be associated to a genetic syndrome that I think that it brought that to the forefront of my mind a little bit more than it had been before. So I think that that was something that I learned or that it reinforced for me.’* (P001)

Some FPs described feeling competent identifying patients at increased risk, but less confident managing them, likely owing to their infrequent presentation in PC. EMR-integrated point-of-care tools offered accessible management pathways based on current screening guidelines, boosting confidence in patient management:

*‘I would say my pre-existing awareness of the importance of identifying genetic conditions was high … my knowledge of what specifically needed to happen with a genetic condition was not high. But that is not uncommon in primary care where you may encounter something very, very infrequently … But knowing they* [point-of-care tools] *were there was helpful, so it added to my sources of information I know I would go to as a valid source of information about genetics*.*’* (P009)

### Success is supported by patient engagement and EMR integration

Having patients complete FHSQ ahead of their appointment, with answers uploaded into the EMR, was described as an efficient and opportunistic way of collecting and/or revisiting FH:

*‘I loved the idea of some of it being done separate from me … it was initiated outside of my doing … those are some of the occasions when I got the information, I wouldn’t have necessarily asked about family history.’* (P008)

FPs shared how annual health exams, where typically FH was updated, are no longer as prevalent, thus some patients may be ‘missed’. This strategy eliminated the need for a dedicated appointment type to update FH:

*‘… it was easy to do it without having to ask all the questions in the middle of a visit. It was actually faster than updating the family history myself. And also, gave us the opportunity to do it at any visit opportunistically, instead of at a dedicated preventative health visit.’* (P001)

### Tailored approaches are needed to improve acceptability

FPs highlighted they were juggling numerous priorities in PC with limited time in a visit. They welcomed efficient strategies to improve patient care; however, some desired further refinement of the strategy for better integration into their workflow. Autonomy was described as a strong facilitative factor; FPs should be able to choose when and for which patients to implement the strategy since FH review rarely leads to urgent follow-up:

*‘… if a patient was coming in for something completely acute, and then you have this strong history, sometimes it felt irrelevant to even bring up in the visit, because there was something else that’s going on. But it was still important information to get and you can never really tell when is the right time to ask these questions, but it just felt easier to ask about it when patients were here for more of a preventive health check.’* (P003)

Workflow integration and routinisation were seen as strengths. Most participants supported continued use but noted the need to avoid adding to FPs’ workload. Suggested improvements included showing only actionable information, allowing FPs to mark FH as reviewed with no updates, using pre-populated FH questionnaires for patients to update, and auto-populating FH into the EMR:

*‘So it has to be able to be incorporated and flow with the current constraints of practice that everyone’s facing. It just — it can’t be viewed as an add-on, or I think it just won’t happen.’* (P009)

Participants said that more patient education would be helpful in enabling patients to understand the rationale for collecting FH and thus present relevant information, thereby increasing efficiency of FH discussion:

*‘Sometimes they would bring up non-pertinent issues … so I think that’s part of educating the patient as well. Well, if you have your great uncle that has those — so it’s not quite as important to us for this purpose as your father or another first-degree relative?’* (P005)

Suggestions to improve the efficiency of the FH questionnaire included adding details such as which relatives were affected and their age at diagnosis.

Some FPs stated that the strategy was best suited for certain patients; for example, those in specific age groups, new to the practice, or not seen recently. Others saw greater value for FPs new to practice, those who didn’t regularly update FH, or lacked easy access to genomics expertise. A key challenge was filtering patient-provided information; involving another team member to review it beforehand was suggested to improve efficiency:

*‘I think we need other healthcare providers doing a lot of background work with patients … who would do more detailed intake assessments that would be comprehensive and include comprehensive family history … given the shortage of available physician time these days, and the demands on patient care, we have to find more streamlined ways, and more efficient ways to do this kind of stuff.’* (P010)

See Supplementary Box S2 for additional quotes supporting the themes.

## Discussion

### Summary

This FH strategy supported improved clinical management through personalised risk-based screening and treatment. Routine collection and review by patients and FPs were central to this process. It also fostered patient-centred conversations and offered insight into psychosocial and family issues. Point-of-care tools boosted FPs’ confidence in managing FH.

Participants appreciated the FH strategy for being patient-driven and completed outside clinical visits. While concerns exist about the accuracy of patient-reported information,^33,34^ studies recommend multi-informant and technology-supported approaches.^34^ A key strength of our strategy was allowing patients to complete the FHSQ outside of appointments, potentially improving the accuracy of FH disclosure.

### Strengths and limitations

Some physicians noted that while the lack of automatic upload into the health summary added time pressure, it also prompted active review of FH, thereby supporting thoughtful clinical engagement. Although time demands were a concern, this manual step helped avoid superficial documentation.

As supported by existing literature^11^ that found FH inquiry did not increase long-term distress in patients, FPs in our study did not report increased patient anxiety, although we acknowledge this is possible; some felt the process could instead raise awareness and encourage healthy behaviours.

Participating clinical practices were located in well-resourced urban and suburban areas, primarily serving educated, middle-class, computer-literate patients. We know that socioeconomic status, including income, is strongly linked to disparities in health outcomes, including lower engagement in preventive care owing to factors such as lack of access, financial constraints, and competing life priorities.^35^ Additionally, those not as comfortable with the use of technology may be less likely to engage in this strategy. As this context may not represent clinics serving more marginalised communities, it would be important to explore and adapt different approaches for diverse PC settings. For example, using paper surveys or allowing patients to complete the FHSQ on a tablet may enhance accessibility.

Additionally, the participating clinics had an affiliation with a practice-based research network, which could foster a culture of innovation and quality improvement. This could have contributed to a more favourable perception of the strategy compared with clinics without such a network connection.

As this study took place during the COVID-19 pandemic, clinic processes and appointment types may have deviated from normal procedures, making it challenging to hypothesise their impact on our findings. Finally, we acknowledge that our small sample size may limit the diversity of opinions and implementation recommendations.

### Comparison with existing literature

This study reinforces existing knowledge that FPs appreciate the need for regular systematic FH collection in PC and its important implications for health,^25,33^ while also highlighting that perceptions of the value of FH strategies may vary among individual FPs.

Barriers to FH collection include time constraints and a laborious process,^7^ while facilitators involve accessible point-of-care tools and routine integration into workflows.^36–38^ Despite FH not being automatically uploaded into the patient’s electronic health summary, our participants valued the integration of FH into the EMR and noted that links to online clinical support tools in the EMR^39^ helped FPs efficiently manage patient-provided information.

The FH strategy can be adjusted based on clinical context and the needs of clinicians and patients. Opinions varied on the type of FH to collect; some prioritised efficiency by gathering only relevant and actionable FH, while others favoured more extensive FH collection, in particular mental health conditions, for understanding patients' psychosocial contexts and overall medical risks. In a qualitative study by Mathers *et al*, FH was widely viewed by GPs as offering valuable insights into the psychosocial context of patients, shaped over time through ongoing relationships. This broader understanding was seen as essential to generalist practice, informing both medical and behavioural aspects of care.^33^

While many FPs in our study described improved communication and more focused discussions prompted by the FH strategy, we also considered potential risks. Specifically, patient-initiated data collection and EMR-mediated prompts may shift the clinical dynamic, potentially altering trust, disrupting workflow, or deprioritising nuanced, in-person dialogue. Although our data did not suggest a negative impact on the therapeutic relationship, and existing literature suggesting patient-initiated data collection and EMR-mediated prompts can enhance patient–physician communication and patient engagement,^40,41^ we acknowledge that automation and task-shifting could, in some contexts, risk fragmenting care. These issues warrant further study, particularly in less-resourced or higher-volume practices.

Most participants found the FH strategy useful but stressed the importance of not adding to FP workload. Strategies for effective implementation might include sending the FHSQ to patients on a rotating schedule, targeting specific patient groups (for example, those new to the practice), and involving the interprofessional team in reviewing results. One randomised controlled trial demonstrated that optimising team roles for screening enhances disease prevention.^42^ Task-shifting might improve efficiency at the cost of relationship building, which was highlighted by participants as a positive unexpected consequence of this strategy. It would be essential for clinical teams to review and adapt the strategy to their specific context and needs.

Our participants noted that FH is typically updated during periodic health exams. With fewer annual visits,^43^ it may be less frequently collected. Our strategy allows patients to share FH independent of appointment type. Administrative data show that higher income and female patients are more likely to receive these visits in Ontario,^44^ raising concerns about inequity and potentially disadvantaging marginalised communities if FH collection is tied to periodic health exams.

Research findings are mixed; however, some studies show that personalised health messages are more effective in motivating individuals to adopt and maintain healthy lifestyles than standardised ones.^45^ Our participants noted that regularly reviewing FH might enhance preventive care and patient outcomes by encouraging greater patient engagement in healthy behaviours.

While many FPs were knowledgeable about inherited cancers, they felt less confident in managing patients with positive FH findings. Literature studying GPs’ views of genetics in clinical practice suggests that GPs believe managing genetic conditions requires specialised, high-level knowledge typically found within the domain of specialists. GPs also express concerns about accuracy when discussing genetics with patients, staying current with evolving evidence, and making appropriate management decisions, such as when to refer patients to clinical genetics.^46–48^ Our strategy, with readily available point-of-care tools, reinforced participants’ knowledge and increased their confidence in discussing these issues with patients. This is in keeping with existing literature that describes that easily available point-of-care tools and routinisation of FH within the workflow will result in improved patient care.^17^

Participants voiced opposing ideas on how the strategy should be implemented, with some recommending only collecting FH at certain appointment types to avoid managing competing priorities within a patient encounter, while others appreciated the opportunistic collection of FH independent of appointment type. Gagné and Deci highlight three pillars that support professionals’ motivation and psychological wellbeing that include autonomy, competence, and relatedness.^49^ Thus, retaining clinician and practice autonomy in deciding how extensive the FH questionnaire and discussion should be are important factors for consideration.

This strategy was likely effective because it addressed many of the concerns and barriers identified by FPs in previous studies on collecting FH,^13,14,33,34,36^ as well as aligning with key CFIR constructs. Important factors to consider when integrating a new strategy into PC workflows include a patient-driven approach, seamless EMR integration with easily accessible resources, and the ability to adapt while preserving FP autonomy.

Regarding CFIR application, we used the CFIR to assess how characteristics of this strategy influenced its effectiveness; the goal of applying the CFIR retrospectively was to map emergent themes to known implementation domains and constructs in order to identify facilitators and barriers and inform future scale-up.

See Supplementary Box S3 for a summary of the application of the CFIR to our strategy.

### Implications for research and practice

One of the highlighted strengths of the strategy was the ability for patients to complete the FHSQ outside of the appointment, which may enable more accurate FH disclosure. This, however, has not been studied, and it would be important to assess in future studies.

Future studies could also assess the effectiveness and acceptability of this strategy when adapted for different contexts and patient populations.

In essence, this patient-driven strategy utilised technology to equip FPs with crucial information that could influence patient management and health outcomes. Importantly, it also addressed many constructs outlined in the CFIR. This approach has the potential to be adopted and adapted across various PC clinics to improve patient care.

In conclusion, FH is crucial for personalised patient management through risk-based screening and treatment. Regular FH collection and review by patients and FPs foster meaningful, patient-centred discussions. This study presents a FH collection strategy, evaluated through an implementation framework, highlighting its strengths and potential optimisations. Key success factors include patient involvement and integration with EMRs. Accessible clinical support tools boost FP confidence in handling FH data. The study emphasises the importance of tailoring FH strategies to specific practice contexts and physician preferences.
